# Impact of Introducing a Controlled-Release Dinoprostone Vaginal Insert for Labor Induction: A Retrospective Single-Center Study in Japan

**DOI:** 10.7759/cureus.53180

**Published:** 2024-01-29

**Authors:** Ryosuke Shindo, Shigeru Aoki, Sayuri Nakanishi, Soichiro Obata, Etsuko Miyagi

**Affiliations:** 1 Perinatal Center for Maternity and Neonates, Yokohama City University Medical Center, Yokohama, JPN; 2 Obstetrics and Gynecology, Yokohama City University Medical Center, Yokohama, JPN

**Keywords:** dinoprostone viginal insert, propess, oxytocin, induction of labor, cervical ripening

## Abstract

Aim: Cervical ripening is commonly performed before oxytocin administration during labor induction in pregnant women with an unfavorable cervix. In Japan, a controlled-release Dinoprostone vaginal insert (CR-DVI) was approved in 2020. Although many studies have compared the mechanical methods of ripening and prostaglandins, few have examined the impact of additional options for labor induction. This study aimed to assess the impact of CR-DVI as an additional option for labor induction in women with an unfavorable cervix.

Methods: In this single-center retrospective study conducted in Japan, 265 participants were divided into two groups: before (January 2018 to May 2020) and after (June 2020 to November 2022) CR-DVI introduction. Before CR-DVI was introduced, hygroscopic dilators were used for all cases instead. On the other hand, after the introduction of CR-DVI, the first choice for cervical ripening was CR-DVI. The CR-DVI was retained vaginally for up to 12 hours after insertion. However, if hyper-stimulation or non-reassuring fetal status was suspected, or if a new membrane rupture occurred, it was removed immediately according to the removal criteria. Oxytocin infusions were used during both periods if needed. We compared delivery and neonatal outcomes between the groups.

Results: The 265 participants were divided into two groups: before (n=116) and after (n=149) CR-DVI introduction. There were no significant differences in maternal characteristics except for the primiparous proportion. CR-DVI was used in 93% of cases after introduction. Hygroscopic dilators also continued to be used; however, their use decreased to about 34%. The vaginal delivery rate was significantly higher after the introduction of CR-DVI than before its introduction (50.9% vs. 66.4%; p=0.01). Multivariable analysis revealed a significantly higher rate of vaginal delivery after CR-DVI introduction. Of the 149 cases in which a CR-DVI was used, 111 (79.9%) were removed before 12 hours. There were no significant differences in neonatal outcomes.

Conclusion: The rate of vaginal delivery was higher after CR-DVI introduction than before its introduction, and adverse pregnancy outcomes did not increase. Therefore, introducing CR-DVI as an option for labor induction may increase the probability of vaginal delivery. Safety can also be ensured by adhering to the removal criteria.

## Introduction

Cervical ripening is commonly performed before oxytocin administration during labor induction in pregnant women with an unfavorable cervix. There are two methods of cervical ripening: pharmacological methods using prostaglandins and instrumental methods using hygroscopic dilators or balloons. Many studies have compared these methods, and a recent meta-analysis reported comparable vaginal delivery rates [1]. However, mechanical ripening methods are reportedly associated with less hyperstimulation and mild neonatal complications [2].

Different from many countries, in Japan, vaginal administration of prostaglandins (PG) was not approved for the induction of labor for a long time, and only mechanical methods were used [3]. Because instrumental cervical ripening procedures are painful to insert, carry the risk of cord prolapse and infection, and require installation techniques, there was a call for the introduction of a PGE2 (Dinoprostone) vaginal formulation, which was the standard method of cervical ripening method overseas. In 2020, a controlled-release Dinoprostone vaginal delivery system (PROPESS®) was approved in Japan for the first time, allowing for pharmacological cervical ripening for labor induction. PROPESS® contains 10 mg of Dinoprostone and releases Dinoprostone at a rate of 0.3 mg/hr for 12 hours. It is a controlled-release Dinoprostone vaginal insert (CR-DVI) that can be removed when needed. Hence, Japanese obstetricians now have an additional option for inducing labor [4]. In our institution, we had only used hygroscopic dilators for women requiring induction of labor with an unfavorable cervix before CR-DVI was introduced. However, after CR-DVI was introduced in 2020, the method of induction of labor for women with unfavorable cervix changed significantly because CR-DVI has become the first choice for cervical ripening methods.

Many studies have compared the mechanical methods of ripening and PGs [1,3,5], and since its introduction in Japan, there have been several reports on CR-DVI [6-10]. However, it has only been a short time since its introduction in Japan, and it cannot be said that it has been fully investigated. In addition, there are few reports comparing pregnancy and delivery outcomes before and after a major policy change from using hygroscopic dilators as the only method of cervical ripening to using CR-DVI as the first choice. This study aimed to examine the impact of the introduction of CR-DVI by comparing pregnancy and delivery outcomes before and after CR-DVI was introduced as a method for labor induction.

## Materials and methods

Study design and participants

This study was conducted per the Declaration of Helsinki guidelines and was approved by the Ethics Committee of Yokohama City University (Approval No. F221000012). Because the study was retrospective, it was not possible to obtain individual written consent from each of the study subjects. We offered study subjects the opportunity to opt-out by publishing a study summary on the institution's website ( https://yokohama-cu.bvits.com/rinri/publish.aspx.) This was a single-center, retrospective study including women who delivered at our hospital between January 2018 and November 2022, underwent cervical ripening procedures to induce labor, and delivered after 37 weeks of gestation. The study setting is Yokohama City University Medical Center, a tertiary care facility with approximately 1000 deliveries annually. Patients with multiple pregnancies, intrauterine fetal death, not cephalic presentation, and premature rupture of the membrane were excluded. We started administering a CR-DVI in June 2020. The participants were divided into two groups: before (January 2018 to May 2020) and after (June 2020 to November 2022) the introduction of a CR-DVI.

Indications for labor induction

Prevention of post-term pregnancy: Cases in which spontaneous delivery did not commence until after 41 weeks and 1 day of pregnancy.

Gestational diabetes/diabetic complicated pregnancy: After 37 weeks and up to 40 weeks of gestation, the obstetrician decides when to start the induction of delivery by evaluating the trend of blood glucose control and cervical ripening.

Hypertensive disorder of pregnancy (HDP): HDP was diagnosed and classified according to the guidelines of the Japan Society for the Study of Hypertension in Pregnancy [11]. Women with preeclampsia, superimposed preeclampsia, severe gestational hypertension, or severe chronic hypertension were induced once the diagnosis was made, either at or after 37 weeks of gestation. Women with non-severe gestational hypertension and chronic hypertension underwent induction of labor by 40 weeks of gestation.

Fetal growth restriction (FGR): FGR was diagnosed when the estimated fetal weight was at least 1.5 SD less than the mean based on Japanese reference values [12]. If the estimated fetal weight was ≤-2.0 SD or -1.5 SD to -2.0 SD with abnormal blood flow (umbilical artery diastolic interruption or regurgitation), the patient was induced promptly at 37 weeks of gestation. In cases of -1.5 SD to -2.0 SD with no blood flow abnormality, the timing of induction for delivery was determined by 40 weeks gestation.

Other: Induction of labor for reasons not listed above.

Cervical ripening

Before the introduction of a CR-DVI, hygroscopic dilators were used to induce delivery in cases of unfavorable cervix (Bishop score of 6 or less). The number of dilators to be inserted was determined at the discretion of the obstetrician. After insertion, the device was retained for at least 12 hours and subsequently removed. After removal, the patients were re-evaluated, and the hygroscopic dilators were reinserted, or oxytocin was administered. In case of non-reassuring fetal status (NRFS), onset of labor, or rupture of the membrane during insertion, the dilators were removed even before the prescribed 12 hours. In these cases, the dilators were not reinserted.

After its introduction, a CR-DVI became the first-choice treatment for cervical ripening, whereas a hygroscopic dilator was used only when a CR-DVI was deemed unsuitable under specific conditions or proved to be insufficiently effective. The criterion for CR-DVI use was a Bishop score of ≤6. The frequency of uterine contractions and the fetal heart rate were monitored using cardiotocography (CTG) during a CR-DVI placement. The device was removed 12 hours after insertion. However, the device was removed before the 12 hours elapsed if the following removal criteria were met: uterine contractions with obvious pain every three minutes for 30 minutes, tachysystole (>5 contractions per 10 min), new or artificial rupture of the membrane, development of fetal dysfunction, and systemic side effects such as nausea, vomiting, and hypotension. A CR-DVI was administered only once. After removal, hygroscopic dilators were inserted, or oxytocin was administered at the discretion of the obstetrician.

Oxytocin use

Oxytocin for labor induction was diluted to 5 U in 500 mL saline and administered at 12 mL/h (2 mU/min). During administration, a CTG was attached, and the dose was increased by 12 mL/hr (2 mU/min) every 30 min after confirming the absence of tachycardia and NRFS, with a maximum dose of 120 mL/hr (20 mU/min).

Background and outcomes

The following data were collected from the medical records. Maternal background: History of vaginal delivery, age at delivery, height, weight before pregnancy, weight at delivery, weight gain during pregnancy, BMI before pregnancy, BMI at delivery, gestational weeks at delivery, and indication for induction of delivery. Delivery outcomes: method of delivery (vaginal or cesarean delivery), cervical ripening, and oxytocin administration. Neonatal outcomes: birth weight, UApH <7.1, Apgar score <7 within 5 min, and neonatal hospitalization.

Statistical analyses

The Wilcoxon rank-sum test was used to compare continuous variables. The χ-square test was used to compare binary variables, with p < 0.05 indicating statistically significant differences. Logistic regression was performed using multivariate analysis for outcomes expressed as binary variables. The adjusted odds ratios (ORs) and 95% confidence intervals (CIs) were calculated using maternal age, delivery experience, BMI at delivery, and weeks of delivery as covariates. All data were analyzed using JMP PRO 16 (JMP Statistical Discovery LLC).

## Results

This study included 265 participants divided into pre-introduction (n=116) and post-introduction (n=149) groups before and after introducing a CR-DVI, respectively. No significant differences were observed in maternal age, pre-pregnancy body size, or weight gain during pregnancy (Table 1). The proportion of primiparous women was 83.6% and 73.8% before and after introduction, respectively; however, the difference was not statistically significant (p = 0.06). Before a CR-DVI was introduced, 100% of the cervical ripening methods were hygroscopic dilators; however, after its introduction, a CR-DVI was used in 139/149 (93.3%) cases. After introducing a CR-DVI, cervical ripening with hygroscopic dilators was used in 51/149 (34.2%) patients. There were differences in the indications for labor induction before and after the introduction of a CR-DVI in cases of GDM/DM and FGR, but not in HDP, prevention of post-term pregnancy, or other indications.

**Table 1:** Maternal background Abbreviation: CR-DVI, controlled release dinoprostone vaginal insert, BW, body weight, BMI, body mass index, IOL, induction of labor, HDP, hypertensive disorder of pregnancy, GDM, gestational diabetes mellitus, DM, diabetes mellitus, FGR, fetal growth restriction. † median (interquartile range); * p-value by χ-square test; ** p-value by Wilcoxon rank-sum test

	Before CR-DVI (n=116)	After CR-DVI (n=149)	p-value
Age, years old^†^	34 (30-37)	33 (30-37)	0.40^**^
primiparas, n (%)	97 (83.6%)	110 (73.8%)	0.06^*^
Height, cm^†^	158 (154-162)	158 (155-162)	0.98^**^
Pre-pregnancy BW, kg^†^	53 (48-62)	56 (50-63)	0.22^**^
Pre-pregnancy BMI, kg/m^2^^†^	21 (19-25)	22 (20-25)	0.31^**^
BW at delivery, kg^†^	65 (60-73)	67 (60-74)	0.34^**^
BMI at delivery, kg/m^2^^†^	26 (24-29)	27 (24-29)	0.35^**^
Gestational weight gain, kg^†^	10 (7-13)	10 (7-13)	0.76^**^
Gestational week^†^	40 (39-42)	40 (38-41)	0.05^**^
Both use (CR-DVI and hygroscopic dilators), n (%)	0 (0%)	41 (27.5%)	-
CR-DVI use, n (%)	0 (0%)	139 (93.3%)	-
hygroscopic dilators use, n (%)	122 (100%)	51 (34.2%)	-
Reason for IOL			
Prevent post-term pregnancy, n (%)	20 (17.2%)	39 (26.2%)	0.06^*^
HDP, n (%)	39 (33.6%)	53 (35.6%)	0.74^*^
GDM/DM, n(%)	8 (6.9%)	34 (22.8%)	<0.01^*^
FGR, n(%)	20 (17.2%)	16 (10.7%)	0.02^*^
Other, n(%)	6 (5.2%)	11 (7.4%)	0.47^*^

Table 2 presents the delivery and neonatal outcomes. The vaginal delivery rate was significantly higher after induction with a CR-DVI than before its use (50.9% vs. 66.4%; p = 0.01). Multivariate analysis revealed a significantly higher rate of vaginal delivery after the introduction, with an adjusted odds ratio of 1.76, 95% CI 1.02-3.06. The rate of concomitant oxytocin use was lower after introducing a CR-DVI than before its introduction (85.3% vs. 69.8%, OR 0.41, 95% CI 0.22- 0.79). There were no significant differences in neonatal outcomes.

**Table 2:** Delivery and neonatal outcomes Abbreviation: CR-DVI, controlled release dinoprostone vaginal insert, aOR, adjusted odds ratio, CI, confidence interval, UA. Umbilical arterial † Adjusted for maternal age, height, body weight, history of vaginal delivery; ‡ median (interquartile range); * p-value by χ-square test; ** p-value by Wilcoxon rank-sum test

	Before CR-DVI (n=116)	After CR-DVI (n=149)	p-value	aOR^†^ (95%CI)
Vaginal delivery, n (%)	59 (50.9%)	99 (66.4%)	0.01^*^	1.76 (1.02- 3.06)
Oxytocin use, n (%)	99 (85.3%)	104 (69.8%)	0.01^*^	0.41 (0.22- 0.79)
Infant birth weight, g^‡^	2950 (2562-3281)	3016 (2677-3359)	0.22^**^	-
Apgar score at 5min <7, n (%)	2 (1.7%)	0 (0%)	0.11^*^	-
UApH < 7.1, n (%)	4 (3.5%)	0 (0%)	< 0.01^*^	-
Neonatal hospitalizations, n(%)	12 (10.3%)	13 (8.7%)	0.65^*^	1.16 (0.50- 2.69)

Table 3 shows the results of the subgroup analysis. Vaginal delivery rates were compared based on previous delivery experiences (primiparous or multiparous) and indications for induced delivery. None of the subgroups had a high rate of vaginal delivery before the introduction of CR-DVI. Significantly higher rates of vaginal delivery after the introduction of CR-DVI were observed for primiparas when the indication for induction of labor was the prevention of post-term pregnancy and for other indications.

**Table 3:** Subgroup analysis Abbreviation: DVI, controlled-release dinoprostone vaginal insert, IOL, induction of labor, HDP, hypertensive disorder of pregnancy, GDM, gestational diabetes mellitus, DM, diabetes mellitus, FGR, fetal growth restriction * p-value by χ-square test

	Before CR-DVI	After CR-DVI	p-value
Primipara	41/ 97 (42.3%)	65/ 110 (59.5%)	0.02^*^
Multipara	18/ 19 (94.7%)	34/ 39 (87.2%)	0.38^*^
Indication for IOL			
Prevent for post-term pregnancy	14/ 43 (32.6%)	26/ 39 (66.7%)	0.01^*^
HDP	24/ 39 (61.5%)	31/ 53 (58.5%)	0.77^*^
GDM/DM	5/ 8 (62.5%)	24/ 34 (70.6%)	0.66^*^
FGR	14/ 20 (70.0%)	9/ 12 (75.0%)	0.76^*^
Other	2/ 6 (33.3%)	9/ 11 (81.8%)	0.04^*^

Of the 139 cases in which a CR-DVI was used, 111 (79.9%) were removed before 12h. The vaginal delivery rates were 67.6% and 67.9% for those whose CR-DVI was removed before 12h and for those whose insertion remained in place for 12h, respectively, with no significant difference (p = 0.98).

Table 4 shows the reasons for a CR-DVI removal in < 12 h and the percentage of vaginal deliveries for each reason. The most common reason for removal was uterine contractions at 3-minute intervals with regular and obvious pain for 30 min (35.1%), followed by tachysystole (30.6%) and NRFS (21.6%). The vaginal delivery rate was the highest for new rupture of the membrane (83.3%), followed by contractions every 3 min (79.5%) and spontaneous prolapse (71.4%). Of the 139 patients who used a CR-DVI, 34 (24.5%) had vaginal delivery without oxytocin.

**Table 4:** Reasons for Removal of CR-DVI and respective vaginal delivery rates Abbreviation: CR-DVI, controlled-release dinoprostone vaginal insert, NRFS, non-reassuring fetal status *  uterine contractions with obvious pain every three minutes for 30 minutes

Reason for removal	Breakdown of reasons for removal (Total number of removed cases= 111)	Vaginal delivery for each reason for removal
uterine contractions every three minutes* n (%)	39 (35.1%)	31/ 39 (79.5%)
tachysystole (>5 contractions per 10 minutes), n(%)	34 (30.6%)	21/ 34 (61.8%)
new rupture of membrane, n(%)	6 (5.4%)	5/ 6 (83.3%)
NRFS, n (%)	24 (21.6%)	13/ 24 (54.2%)
other, n (%)	1 (0.9%)	0/ 1 (0%)
spontaneous prolapse, n(%)	7 (6.3%)	5/ 7 (71.4%)

## Discussion

The present study showed higher rates of vaginal delivery and lower rates of concomitant use of oxytocin after the introduction of a CR-DVI compared to those before its introduction. Additionally, no increase was observed in adverse pregnancy outcomes after the introduction of a CR-DVI by removing it by the removal criteria.

The results of the latest Cochrane reviews and network meta-analyses suggest that the effects of mechanical ripening methods and PG formulations are the same [1,2]. Regarding mechanical ripening methods, there are many reports on balloons but only a limited number on hygroscopic dilators. We have previously compared the pregnancy and delivery outcomes of 17,363 primiparas undergoing mechanical ripening using the Japanese Society of Obstetrics and Gynecology database and found no difference in vaginal delivery rates between hygroscopic dilatation materials and balloons [13]. A randomized controlled trial with the primary outcome of non-inferiority of hygroscopic dilators to balloons has also been reported [14]. Thus, these results suggest no significant difference in the rate of vaginal delivery between hygroscopic dilators and PGE2.

In contrast, the present study showed a high rate of vaginal delivery after the introduction of a CR-DVI. We hypothesized that this was because some patients who did not achieve vaginal delivery with conventional mechanical cervical ripening and oxytocin had poor sensitivity to oxytocin, and Dinoprostone/ PGE2 was effective in these cases. Notably, in the present study, oxytocin use decreased significantly after the introduction of CR-DVI, and 24.5% of the patients who used CR-DVI had a vaginal delivery without oxytocin. This effect may be due to the uterine contractile effects of PGE2. Previous reports have shown that the use of PGE2 is associated with decreased oxytocin use [15,16]. It is possible that some cases in which the effects of PGE2 initiated labor without the need for oxytocin included those with a poor response to oxytocin. Therefore, the availability of vaginal PGE2 as an option for induction of labor may have contributed to the increase in the vaginal delivery rate.

This study showed no differences in adverse pregnancy outcomes before and after introducing a CR-DVI. According to a recent Cochrane study, cervical ripening with PGE2 is associated with greater hyperstimulation and adverse events in neonates than balloon ripening [2]. However, this may be because PGE2 formulations were not categorized into removable and non-removable formulations. The removable formulation can be safely used by adhering to the removal criteria. The results of the present study revealed that a CR-DVI was removed in < 12 hours in approximately 80% of the cases because of compliance with the removal criteria. Although the rate of vaginal delivery was lower when removal was due to NRFS than when it was due to other reasons, there was no difference in the overall rate of vaginal delivery between patients whose CR-DVI was removed during the procedure and those who retained the drug for 12 hours. This result suggests that appropriate use, in compliance with the removal criteria, can minimize adverse events without decreasing efficacy. While a few studies comparing removable and non-removable preparations have shown no difference in the frequency of hyperstimulation in either case [17,18], no large-scale studies have addressed the prognosis of infants. Notably, the results of this study showed no adverse effects on neonatal outcomes after the introduction of a CR-DVI.

This study had some limitations. The most significant limitation was the inability to collect data on bishop scores accurately. We could only obtain a score of less than 6 from the medical records. Next, because this was a single-center retrospective study, there was a bias in the background of the patients included. Specifically, there were differences in the indications for labor induction before and after the introduction of a CR-DVI. However, the subgroup analysis revealed a higher rate of vaginal delivery after the introduction of a CR-DVI, even when compared based on the indications for induction. In addition, while the institution had specific guidelines for determining cervical ripening procedures and the timing of labor induction, the final decision was at the discretion of the obstetricians, reflecting a more flexible institutional policy. The decision to carry out a cesarean section was also left at the discretion of the physician. Furthermore, the small sample size prevented accurate comparison of serious neonatal complications, which are infrequent. Therefore, a larger multicenter study is required to generalize our findings.

## Conclusions

The rate of vaginal delivery was higher after the introduction of a CR-DVI as an option for labor induction than before its introduction. Moreover, the incidence of adverse pregnancy outcomes did not increase. Therefore, introducing a CR-DVI as an option for labor induction, in addition to the use of oxytocin and a hygroscopic dilatant alone, may increase the rate of vaginal delivery. Safety can also be ensured by adhering to the removal criteria.
